# Stable *in vitro* fluorescence for enhanced live imaging of infection models for *Batrachochytrium dendrobatidis*

**DOI:** 10.1371/journal.pone.0309192

**Published:** 2024-08-29

**Authors:** Rebecca J. Webb, Andrea L. Vu, Lee F. Skerratt, Lee Berger, Francisco De Jesús Andino, Jacques Robert

**Affiliations:** 1 Faculty of Science, One Health Research Group, University of Melbourne, Victoria, Australia; 2 Department of Microbiology and Immunology, University of Rochester Medical Center, Rochester, New York, United States of America; Shiv Nadar University, INDIA

## Abstract

Realistic and modifiable infection models are required to study the pathogenesis of amphibian chytridiomycosis. Understanding the mechanism by which *Batrachochytrium dendrobatidis* (Bd) can infect and kill diverse amphibians is key to mitigating this pathogen and preventing further loss of biodiversity. *In vitro* studies of Bd typically rely on a tryptone based growth media, whereas the recent development of a kidney cell-line infection model has provided a more realistic alternative, without the need for live animals. Here we use expression of a fluorescent reporter to enhance the *in vitro* cell-line based growth assay, and show that transformed Bd cells are able to invade and grow in an amphibian kidney epithelial cell line (A6) as well as in a new system using a lung fibroblast cell line (DWJ). Both Bd and host cells were modified to express reporter fluorescent proteins, enabling immediate and continuous observation of the infection process without the need for destructive sampling for fixation and staining. Plasmid DNA conferring hygromycin resistance and TdTomato (RFP) expression was delivered to Bd zoospores via electroporation, and continuous antibiotic selection after recovery produced stable fluorescent Bd transformants. Host cells (A6 and DWJ) were transfected before each assay using lipofection to deliver plasmid DNA conferring green fluorescent protein (GFP) and containing an empty shRNA expression cassette. Bd RFP expression allowed easy localisation of fungal cells and identification of endobiotic growth was assisted by host GFP expression, by allowing visualization of the space in the host cell occupied by the invading fungal body. In addition to enabling enhanced live imaging, these methods will facilitate future genetic modification and characterisation of specific genes and their effect on Bd virulence.

## Introduction

Pathogenic fungal infections in humans and wildlife are of increasing concern [1]. The amphibian chytridiomycetes *Batrachochytrium dendrobatidis* (Bd) and *B*. *salamandrivorans* (Bsal) are unusual in that they are the some of the only chytrid species known to infect vertebrates. Both emerged as pathogens after escaping from Asia [2]. Bsal predominantly infects caudata (salamanders and newts), and has decimated some European populations, and further severe impacts are predicted if it spreads to salamander hotspots such as North America [3]. In contrast, Bd has already spread globally and is regarded as the world’s worst wildlife pathogen [4], having driven at least 90 species to extinction [5]. The impact of Bd is partly due to its remarkably large host range, being able to infect over 700 amphibian species [6].

Chytrids are characterised by their unwalled motile zoospores. Zoospores of Bd infect amphibian skin, invading via germ tube [7], before developing into zoosporangia within epidermal cells [8]. Zoosporangia asexually produce a new generation of infectious zoospores that are released via a discharge tube. High infection burdens develop into chytridiomycosis, a disease characterised by abnormal skin, including increased sloughing, ulcers and erythema [9]. In Bd infections, the skin disruption can lead to loss of homeostasis via electrolyte depletion, resulting in cardiac failure and death [10,11].

Prior to the detection of Bd, the Chytridiomycota were a relatively understudied group. Understanding aspects of Bd virulence such as host detection, immune evasion and zoosporangia maturation could inform targeted interventions. For many years *in vitro* studies of Bd have been limited to using a tryptone based growth media [12] that is not representative of the host environment, and hence limits our ability to fully understand virulence factors. For example, Bd zoosporangia in tryptone cultures are larger [8] and develop rhizoids, which are rarely observed during host invasion. In the host, the timing of Bd maturation matches epidermal cell differentiation and discharge tubes protrude to the skin surface [8]. Gene expression also changes in culture, with many putative virulence genes only switched on during infection [13–15]. Recently, Bd *in vitro* culture methods have been greatly improved by the development of an amphibian cell line infection model [16], which has been used to compare virulence of Bd isolates [17]. Zoospores will encyst, develop germ tubes, and invade the *Xenopus laevis* kidney A6 cell line. However, visualisation of infections involves destructive sampling and fixation followed by antibody staining. Creating Bd isolates that express fluorescent proteins would allow non-invasive, real-time observation of host cell infection. This is a common strategy in the study of other fungal pathogens, for example transformation of the phytopathogens *Ustilaginoidea virens* [18], *Fusarium oxysporum* [19] and the human pathogen *Candida albicans* [20] to express green fluorescent protein (GFP) has enabled tracking of infection *in planta* and *in vivo* murine models. However, GFP expression does not produce measurable fluorescence in the chytrid *Spizellomyces punctatus*, possibly due to protein misfolding [21]. Therefore, we sought to transform Bd with a construct conferring a red fluorescent protein (RFP), tdTomato, to enable easy visualisation of Bd cells. In addition, transformation of host cells with a contrasting fluorescent protein will further enhance visual analysis and location of the invading Bd relative to cell contents. Here we expand the utility of the *in vitro* cell growth assay technique using fluorescent expression in both the Bd and host cells (A6) to enable non-invasive live imaging and continuous observation of the infection process. To expand this model for flexibility and customisation, we validated this approach in a second cell type, using *X*. *laevis* lung fibroblasts (DWJ).

## Materials and methods

### Plasmids

**Fig 1:**
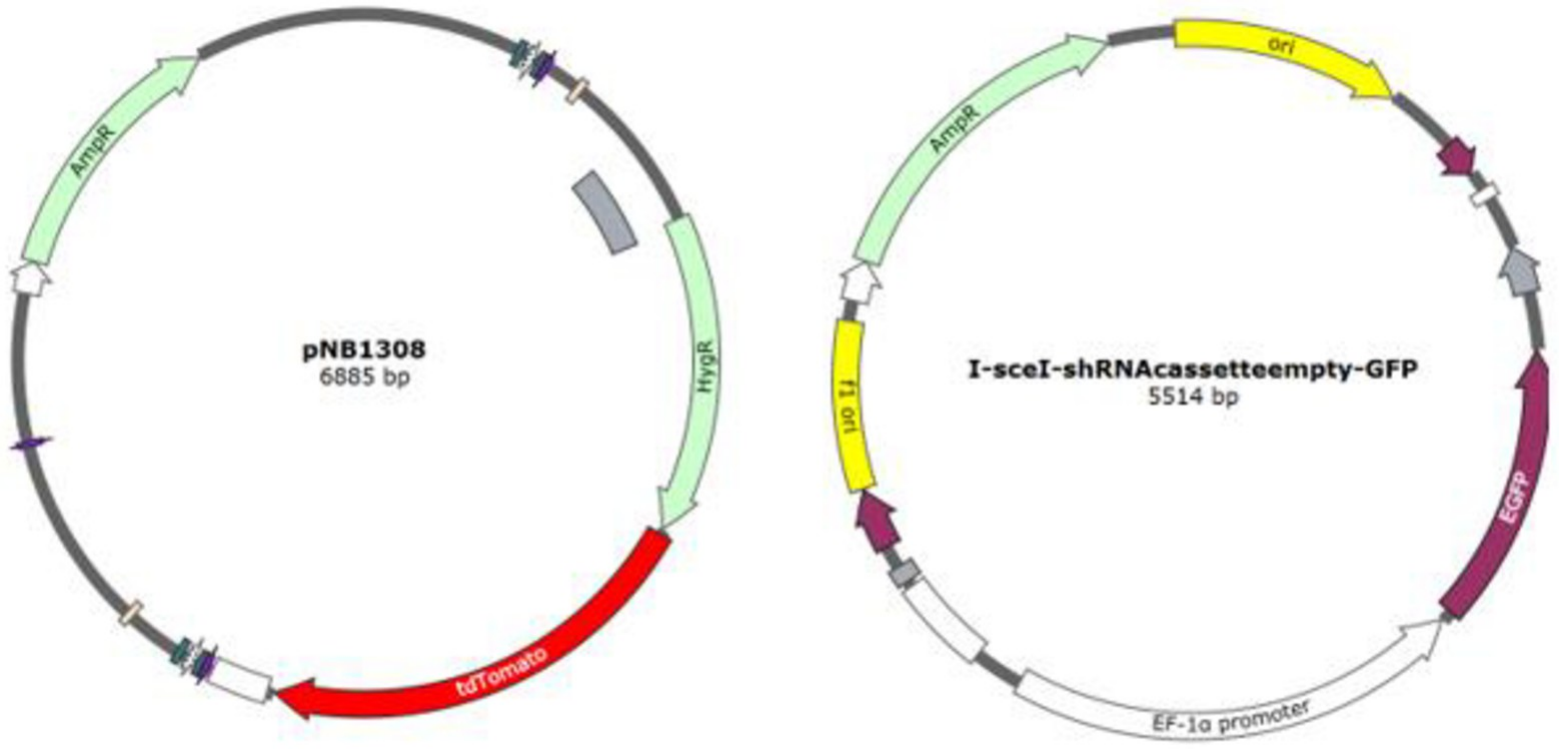
Vector maps for plasmids used in this study. pNB1308 was used for Bd transformation, and I-sceI-shRNAcassetteempty-GFP used for amphibian cell line transfection.

Plasmid pNB1308 contains a gene for tdTomato red fluorescent protein fused to a hygromycin resistance gene, under the control of a *Spizellomyces* Histone 2B promoter in a pUC19 background, created using standard restriction digest/ligation cloning (Fig 1). Plasmid I-sceI-shRNAcassetteempty-GFP contains GFP under the control of an EF-1α promoter [22] (Fig 1). Both plasmids were purified from *E*. *coli* using PureYield plasmid Maxiprep system (Promega), sterilised by ethanol precipitation, and resuspended in sterile water at ~1 μg/μL.

### Bd culture and transformation

*Bd* isolate JEL 197 was maintained in a tryptone, gelatin hydrolysate and lactose (TGhL) media as per standard protocols [23]. Synchronised zoospores were transformed with plasmid pNB1308 as follows. TGhL media from mature flasks was removed and the zoosporangia monolayer incubated with fresh TGhL for ~2 h [12] to allow zoospore release. The resulting zoospore solutions were syringe filtered using a sterile 10 μm isopore filter (Millipore) to exclude zoosporangia [24], concentrated by centrifugation at 2500 x *g* for 5 min at RT, and re-suspended in sterile Petri’s solution (0.25 mM CaCl_2_, 1 mM MgSO_4_, 1 mM KH_2_PO_4_, 0.8 mM KCl) [25] to a final concentration of 2 x 10^7^ zoospores per mL. Zoospores (200 μL) were transferred to a 2 mm cuvette (Bio-Rad) with 0.2 μg/μL DNA, chilled on ice (5 min) before electroporation using a Bio Rad electroporator set at 420V, 50 μF, and 1000 Ω [25]. After electroporation, zoospores were briefly chilled on ice (1min) before gentle transfer to 25cm^2^ culture flasks containing 10 mL TGhL. After overnight recovery at 20°C, the TGhL media was removed and replaced with fresh TGhL with 10 μg/mL hygromycin B (Sigma), which is well above the MIC for wildtype Bd [26]. Transformed *Bd* cells were continuously maintained in TGhL + hygromycin and passaged weekly. After 3–4 generations, a single clone was generated by spreading zoospores on a TGhL + hygromycin agar plate and selecting a single colony. Pure zoospore suspensions of transformed Bd and wildtype Bd were plated in TGhL (without hygromycin) to monitor for any off-target changes in growth.

### Amphibian cell culture and transformation

The A6 cell line was generated from an outbred *Xenopus laevis* kidney [27] and DWJ cell line from a J-frog *X*. *laevis* lung [28,29]. Cells were maintained in amphibian serum free (ASF) medium containing 10% FBS, supplemented with penicillin, streptomycin and kanamycin [30,31] in 75cm^2^ flasks at 27°C. Cells were transfected with I-sceI-shRNAcassette-empty-GFP using lipofection as follows: Cells were detached by incubation with 0.25% trypsin (2.5 μg/ml) for 5 min at 27°C, suspended in 5 ml ASF, and spun at 1000g for 5 min at RT. The supernatant was removed, and cells resuspended in fresh ASF media, and plated in 24 well plates at 1 x 10 ^5^ cells per well. After incubation at 30°C for 3 h, ASF media was removed and replaced with 500 μL of solution A (70% L15, 20% water, 10% FBS) [16] for 1 h. Following the manufacturers’ instructions, 2 μL Lipofectamine 2000 (Thermo Fisher) and 0.8 μg plasmid DNA in 100 μL Opti-MEM (Gibco) was added to each well and incubated overnight at 27°C. Transfection success was calculated as the proportion of GFP expressing cells per field of view.

### *In vitro* infection of A6 and DWJ cells with transformed Bd

To establish the feasibility of including transformed Bd in the *in vitro* cell infection protocol developed by Verbrugghe *et al* 2019 [16], we followed their methods with the following modifications. Transformed and wildtype zoospores were collected from mature culture flasks as described above, centrifuged and resuspended in solution C (20% L15, 77.5% water, 2.5% FBS) to a final concentration of 1 x10^6^ zoospores per mL. Excess media was gently removed from the host cells and replaced with 500 μL zoospore solution at 20°C. After allowing 1 hr for the zoospores to encyst, excess media was removed and replaced with solution B (40% L15, 55% water, 5% FBS) [16] for 1hr, then replaced with solution A, sealed with parafilm and incubated at 20°C, 25°C and 27°C. Zoospores in solution C were also added to wells without host cells, incubated for 1 hr, replaced with solution B, then incubated in solution A or TGhL for comparison. The effect of transformed and wildtype Bd on host cells was observed daily using light microscopy. The development of transformed Bd was monitored using an EVOS FL Digital Inverted Fluorescence Microscope (Life Technologies) to take merged images using red (Bd) and white light. Once optimised the infection procedure was repeated using host cells transformed to express GFP, and merged white, red and green images were captured.

## Results

**Fig 2:**
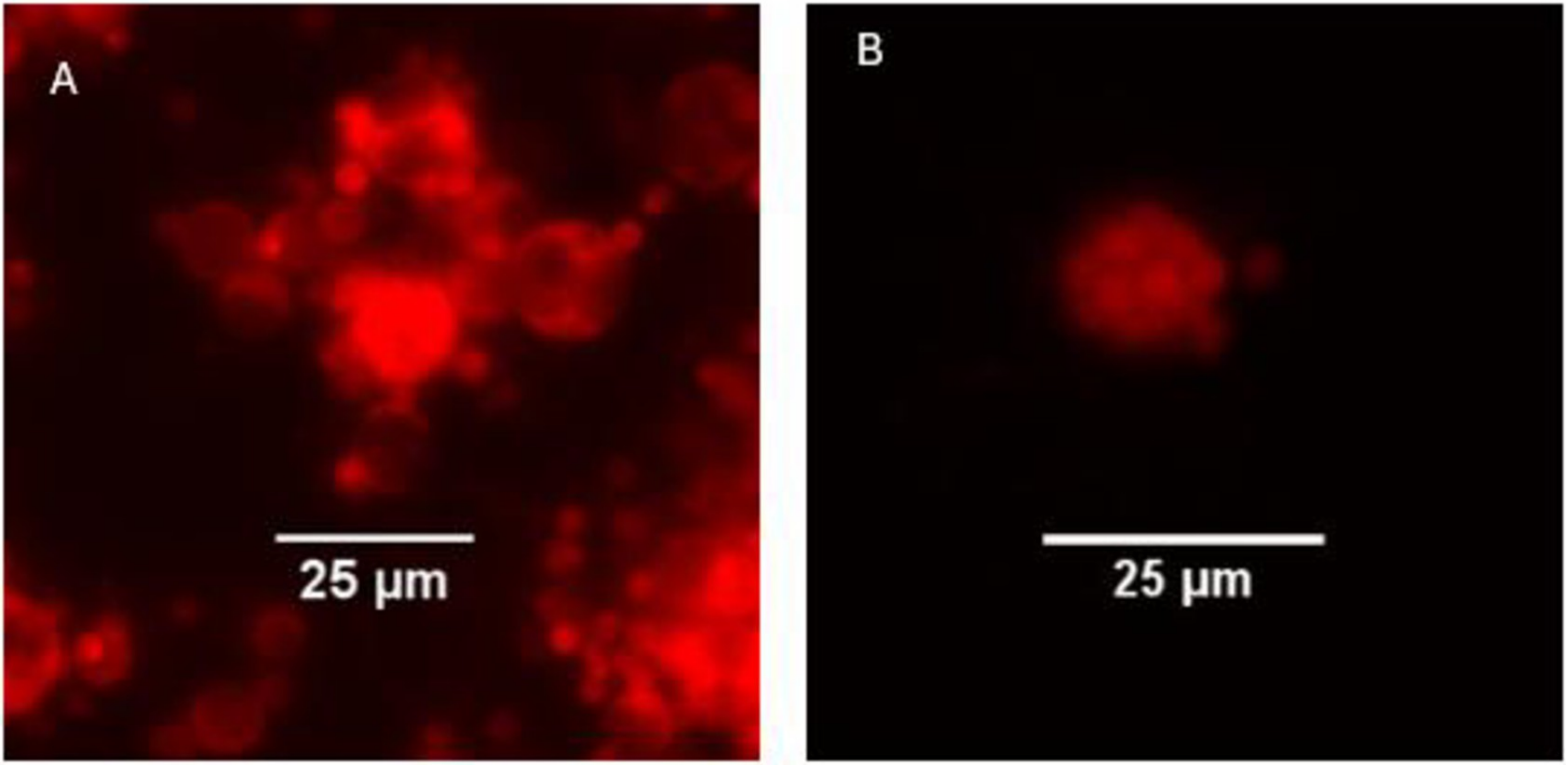
Transformed Bd "Tom-Bd" displaying bright red fluorescence. A = mixed population of different life stages. B = Zoospores developing inside a zoosporangium.

**Fig 3:**
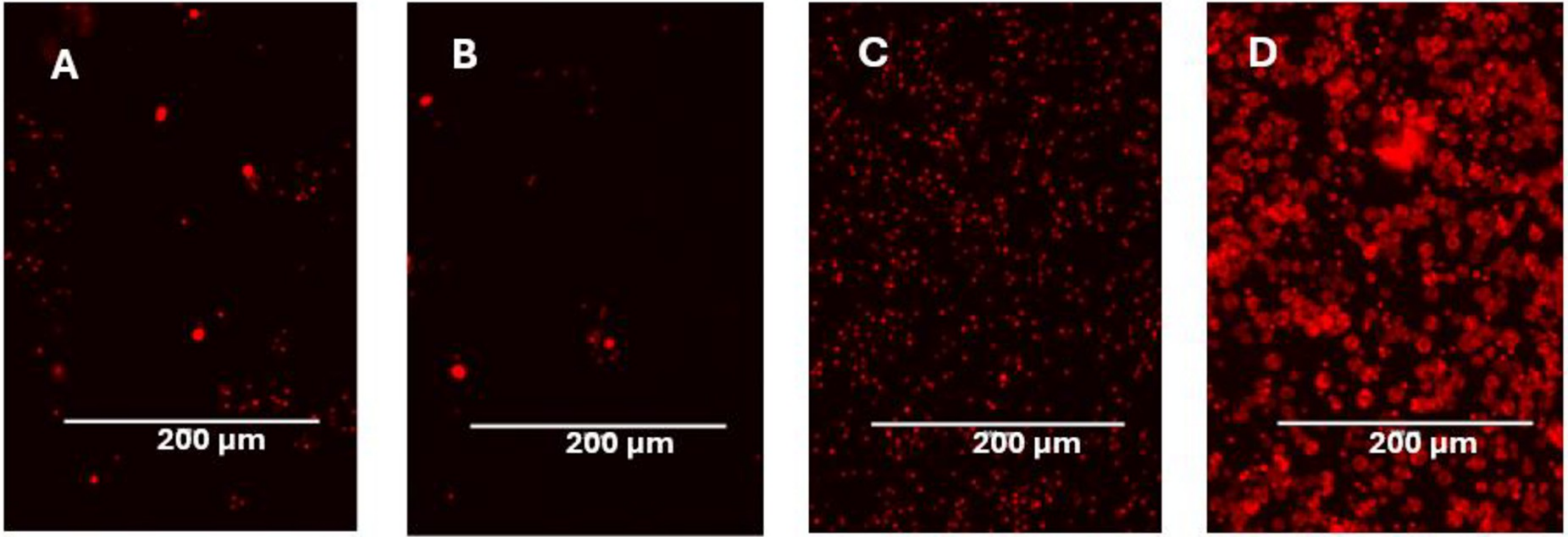
Growth of transformed Bd (Tom-Bd) 24 hr after inoculation in host cells compared to host cell media and Bd media. Bd growth was slower in A6 and DWJ host cells compared to TGhL media. Bd zoospores did not grow in host cell media alone. A = A6 cells, B = DWJ cells, C = Cell media, D = TGhL.

Using hygromycin selection and subcloning, we created a stable Bd strain (Tom-Bd) displaying hygromycin resistance and bright tdTomato fluorescence (Fig 2). Cultures of Tom-Bd displayed a growth rate consistent with wild type Bd, with simultaneous zoospore release when grown in TGhL (S1 Fig). When used in the *in vitro* cell infection assay, both wild type and Tom-Bd zoospores were motile in solution C, but encysted quickly regardless of host cell presence. However, Bd maintained in cell media (solution A) without host cells did not grow after encysting, indicating that host cells provided the nutrients for Bd maturation (Fig 3). Both wild type and Tom-Bd produced comparable cytopathic effects on host cells when observed by light microscopy (S2 Fig). The growth of both wild type and Tom-Bd in host cells differed markedly from growth in TGhL; Bd density in host cells was < 10% compared to TGhL (Fig 3), and zoosporangial growth was also slower in host cells compared to TGhL (Fig 3). Wild type and Tom-Bd grown in TGhL released zoospores as early as 48 hours after encystation, whereas zoospore release was only observed after 4 days on host cells.

**Fig 4:**
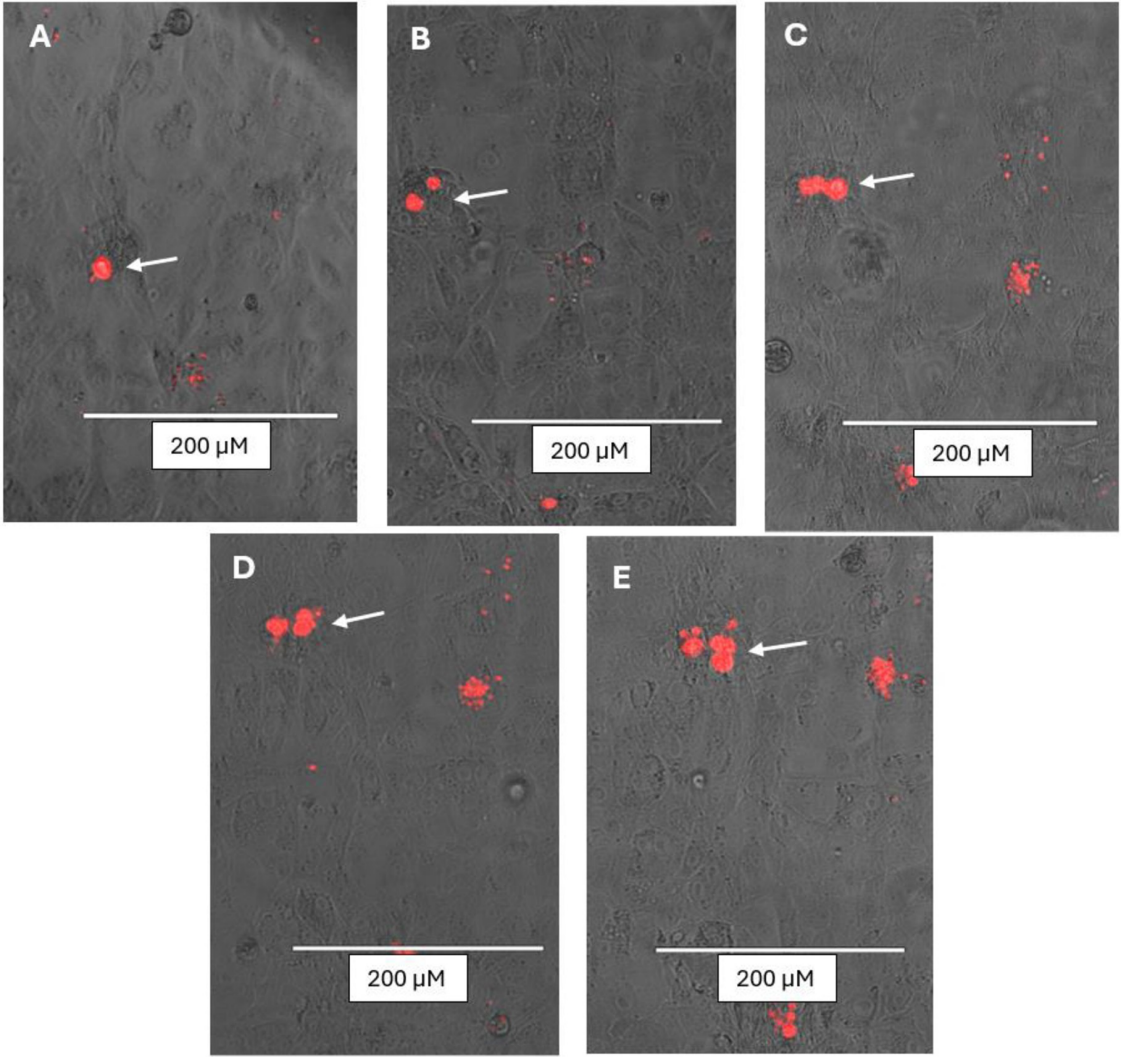
Time series images of Tom-Bd development in DWJ cells. A = 48 h, B = 72 h, C = 96 h, D = 120h, E = 144 h. The same location (arrow) was imaged over multiple days.

**Fig 5:**
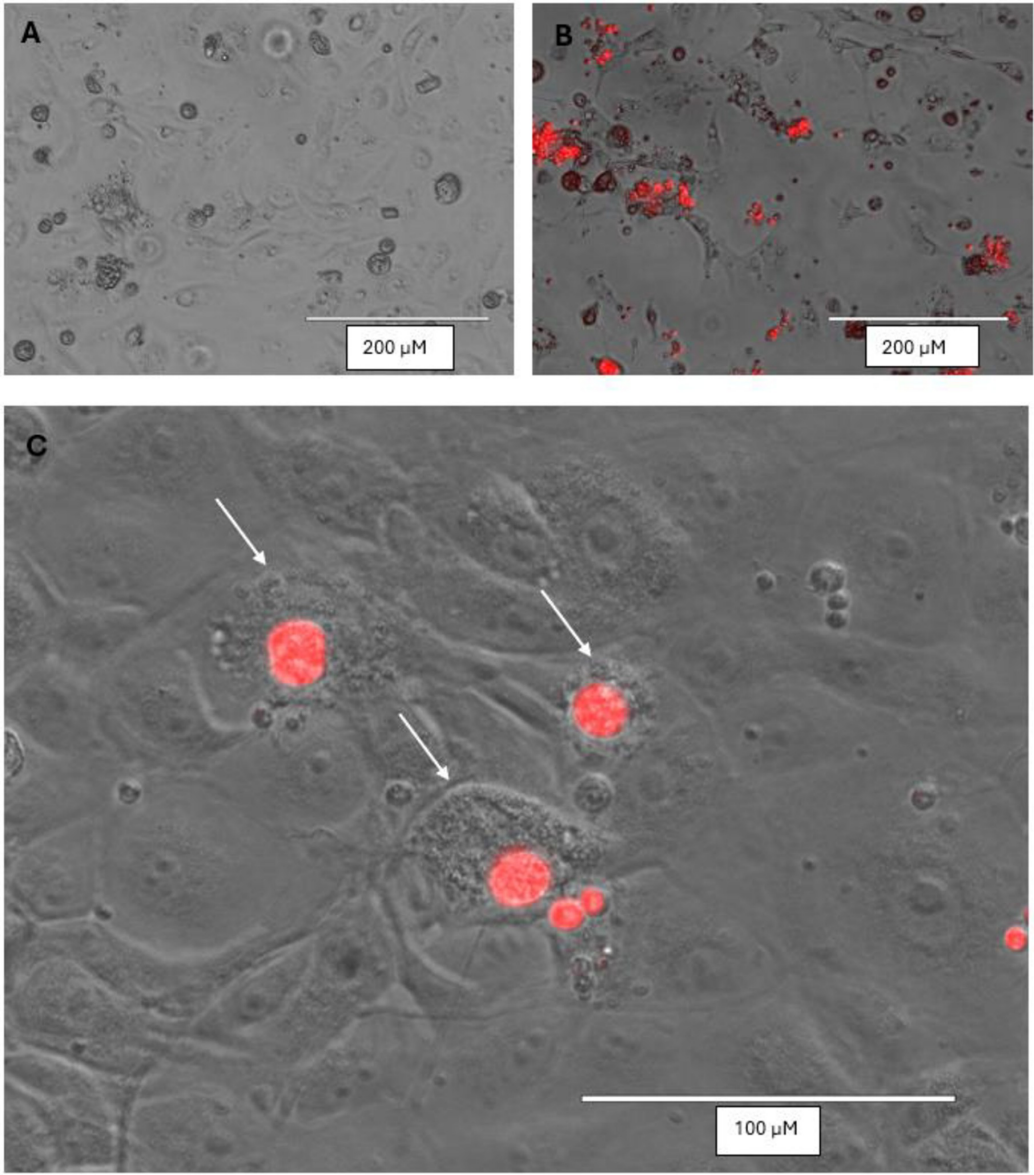
Effect of Bd infection on host cells. Merged images at 120 h post infection; healthy, uninfected A6 cells (A) and infected A6 cells showing cytopathic effects (B) (Scale bar = 200 μM). Higher magnification of degradation (white arrows) around Bd cells in A6 (C) cells at 96 h post infection (scale bar = 100 μm).

Due to their bright red fluorescence, the interaction between Tom-Bd and host cells can be observed immediately, enabling observation of the same cells repeatedly over many days to monitor growth and infection outcomes (Fig 4). We tested the *in vitro* infection system at three different temperatures; 20°C (Bd optimum), 24°C (intermediate), and 27°C (host cell optimum). Growth of Tom-Bd was higher at 19°C and 24°C, with fewer mature zoosporangia at 27°C (S3 Fig). Both types of host cells were negatively affected by Bd infection as evidenced by the area of cytoplasmic degradation around invading Bd cells, cytopathic effects on infected cells compared to uninfected (Fig 5), as well as the observation of detached dying host cells containing internal Bd (Fig 7). These pathogenic effects align with those described by Verbrugghe *et al* 2019, suggesting that transforming Bd to express tomato fluorescent protein does not compromise the applicability of the *in vitro* infection assay.

**Fig 6:**
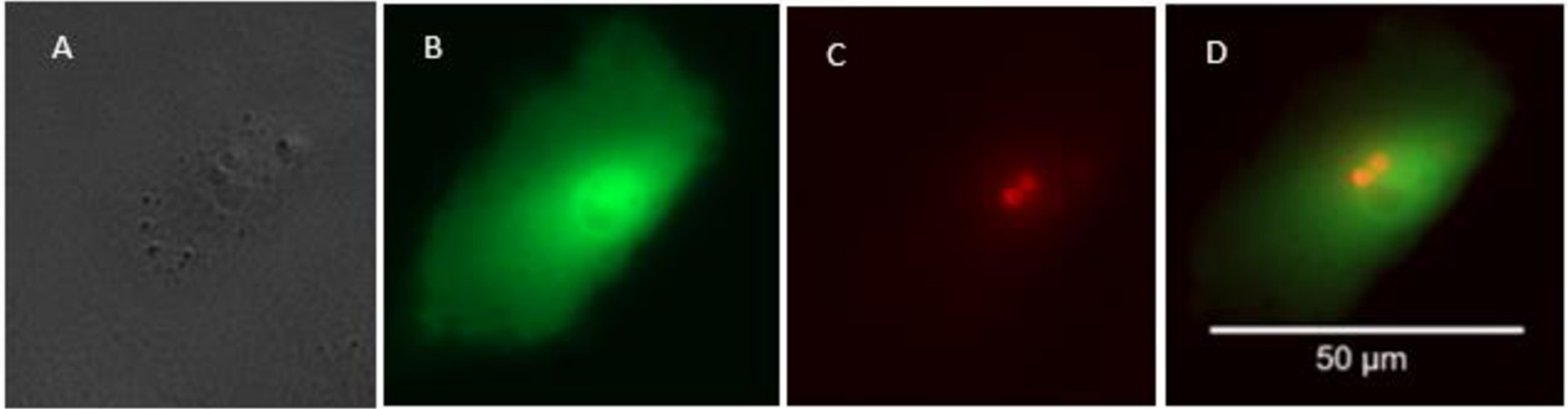
Early Bd infection of an amphibian cell in vitro. GFP expressing DWJ fibroblasts 2 h after exposure to Tom-Bd zoospores. Images taken with transmitted light (A), GFP filter (B), RFP filter (C) and merged (D).

To further enhance in situ observation of the infection process, the host cells were transformed to express GFP. The I-sceI-shRNAcassetteempty-GFP vector does not contain an antibiotic selective marker, therefore we transformed host cells the day before infection. Transformation success of A6 host cells was about 10%, which is typical for this cell type [32], whereas DWJ cells were easier to transfect, and resulted in approximately 20% success rate. GFP expression persisted for the duration of the infection experiment, and the mosaic GFP expression allowed clear definition of host cell boundaries (Fig 6).

**Fig 7:**
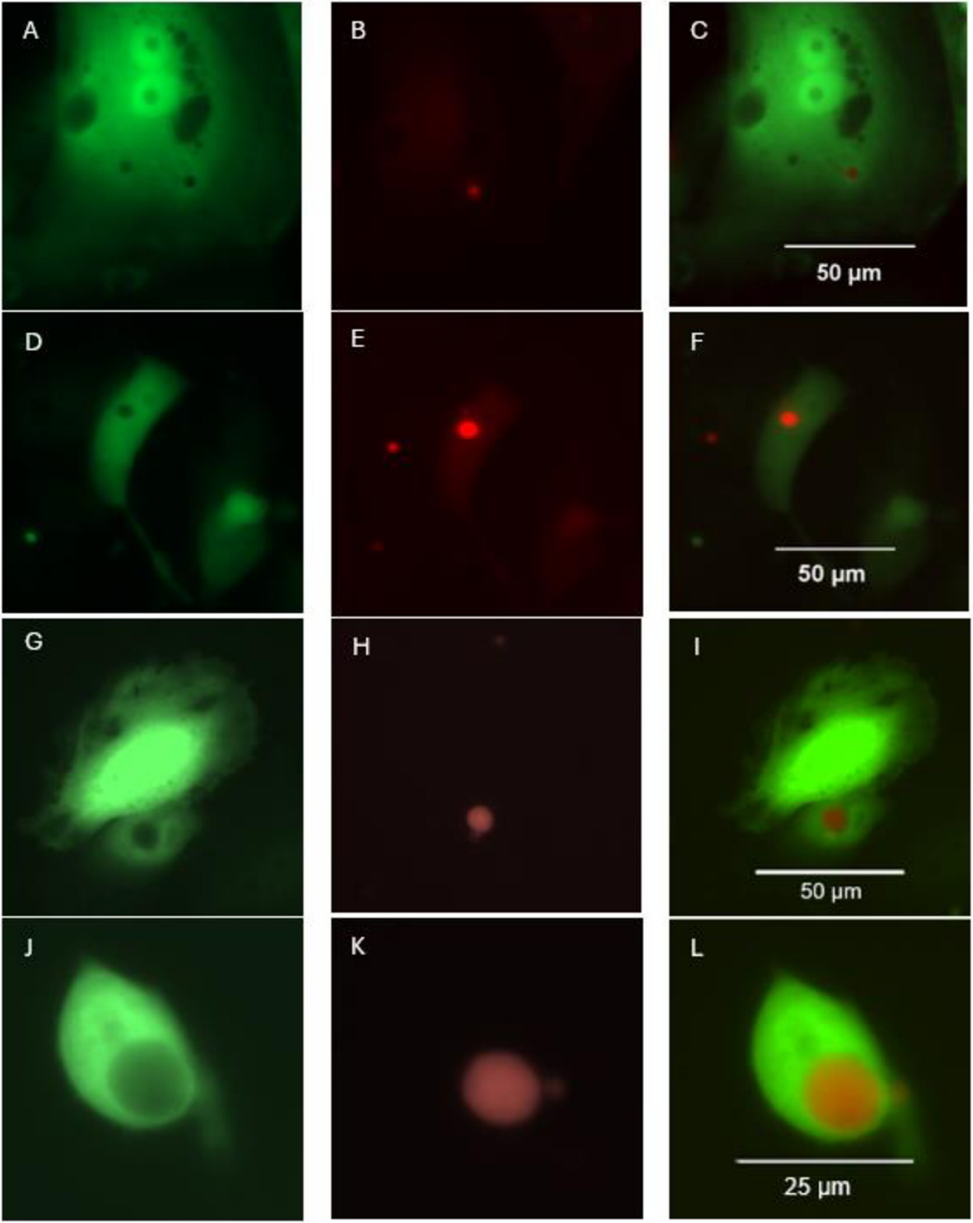
GFP expression assists identification of endobiotic growth, by showing gaps in the host cell. A-C = DWJ cell 8 h after Tom-Bd infection (GFP, RFP, merged), D-F = DWJ cell 24 h after Tom-Bd infection (GFP, RFP, merged), G-I = A6 cell 96 h after Tom-Bd infection (GFP, RFP, merged). L-J = Detached, dying A6 cell 96 h after Tom-Bd infection (GFP, RFP, merged).

We observed both epibiotic and endobiotic Bd growth in both host cell types. Identification of endobiotic growth was assisted by host GFP expression, which allowed visualization of the gap in the host cell occupied by the invading fungal body (Fig 7).

## Discussion

We have enhanced an *in vitro* infection model for research on chytridiomycosis by enabling visualisation of all growth stages via genetic modification of both Bd and amphibian host cells (A6 & DWJ) to express fluorescent proteins. This method enabled immediate and continuous observations of the infection process, allowing tracking of individual cells in real time without interference. Generating stable transformed Bd (Tom-Bd) overcomes the time-consuming process of fixation, permeabilization and immunohistochemical staining of the cells for visualisation using different replicates for each time point. The use of Tom-Bd also enabled direct observation of internalised Bd in detached host cells, which would have otherwise been lost in the washing steps, and therefore avoids a possible underestimation of cytopathic effects. In this work we transformed Bd with a fluorescent marker fused to an antibiotic selective marker to produce stable transformants which have continued to maintain fluorescence over many generations. Development of this transformation protocol is a key step in future functional genetic studies, previously thought to be a difficult goal [33]. Our construct together with the enhanced *in vitro* assay provides a new approach to modify the expression of putative virulence genes and characterize their effects. Our inclusion of GFP expressing host cells allowed for clear definition of cell boundaries, and easy identification of endobiotic Bd growth, especially during the later stage of infection when mature Tom-Bd cells produce a large gap in the host cell. At the early stage of infection, observation of the small Tom-Bd structures is possible, but can be further enhanced by the use of a high powered inverted microscope or confocal microscopy. Cell fluorescent expression is not expected to interfere with antibody recognition or chitin; therefore, the staining techniques developed by Verbrugghe et al 2019 could also be used in addition to in situ fluorescence for observing specific structures. The extreme brightness of the fluorescence may reduce fine scale resolution, so for further work such as visualising fungal attachment and invasion, there may be advantages in combining various stains for examination under different conditions.

An earlier study demonstrated that Bd can complete its life cycle in *X*. *laevis* A6 kidney epithelial cells [16], and here we confirm that lung fibroblasts (DWJ) of this species can also be used. We successfully transfected both cell types with a GFP expressing empty shRNA vector as proof of concept, but plan to insert functional shRNA to target Bd virulence genes and host resistance genes to assess their function. Previous studies have found *X*. *laevis* A6 cells inherently difficult to transfect, with efficiencies below 6% [32]. We found DWJ cells produced higher transfection success, thus this cell type will be useful for experiments requiring host cell genetic modification. Further optimisation of transfection success rates could employ co-transfection with selectable markers, or the use of transduction as an alternative. As the optimum growth temperature of Bd is lower than that of the host cells, we assayed the *in vitro* infection system at temperatures between 20–27°C. Tom-Bd zoosporesdeveloped into zoosporangia at all three temperatures, but growth was higher at the intermediate temperature of 24°C (S3 Fig). Aside from temperature, this assay also requires cell media that supports both host and Bd. We used a more dilute inoculation solution than the original assay [16], but still found that zoospores encysted rapidly (often in unfavourable locations outside of host cells), perhaps explaining the low cell density compared to TGhl media (Fig 4). Future work should investigate alternative inoculation solutions that prolong zoospore motility and support host cell health. Previously, the investigation of Bd virulence factors often relied on the use of live animals, either live amphibians, harvested amphibian tissue [34] or alternative model species such as zebrafish [35]. This enhanced fluorescent *in vitro* assay avoids animal experimentation and allows real time observation of realistic Bd invasion. Although infections within amphibian stratified and keratinising epidermis are different to these cell culture monolayers, similar pathological changes were observed during Tom-Bd infection such as cell contraction [8], and the sporangial morphology was similar to the parasitic growth form in that rhizoids were rare. We confirmed that Bd was utilising host cells as a nutrient source, but found that Bd grew slower in host cells compared to TGhL culture media. Bd also grew at a much lower density in host cells, with many zoospores encysting, but only a fraction developing into mature zoosporangia (Fig 4). Hence, experiments using traditional TGhL assays may be overestimating the growth rate of Bd compared to host infections.

In summary, through the stable transformation of Bd and delivery of a GFP shRNA expression cassette to A6/DWJ host cells, our modification of an established *in vitro* infection model facilitates real-time observation within the cellular environment, thereby offering a platform for the investigation and manipulation of virulence genes in subsequent studies.

## Supporting information

S1 Fig Growth of wildtype and transformed (Tom-Bd) Bd after 72 h in TGhL Growth rate and zoospore release was similar between original wildtype and transformed Bd. A = Wild type, B = Tom-Bd. Scale bars = 200 μm.
(DOCX)

S2 Fig Growth of wildtype or transformed Bd in host cells. **Both wildtype and Tom-Bd produced similar effects on host cells at 96 h post infection.** A = Uninfected A6 cells, B = WT infected A6 cells, C = Tom-Bd infected A6 cells. D = Uninfected DWJ cells, E = WT infected DWJ cells, F = Tom-Bd infected DWJ cells. Scale bars = 200 μm.
(DOCX)

S3 Fig Growth of Tom-Bd in DWJ cells at different temperatures. A *=* 20°C, B *=* 24°C, C *=* 27°C. D *=* Number of mature zoosporangia in DWJ cells after 120 h incubation at different temperatures. Mature zoosporangia were counted per field of view (n *=* 15) at 20x.
(DOCX)
