# Supplementary material for: Stable in vitro fluorescence for enhanced live imaging of infection models for Batrachochytrium dendrobatidis
Source: PLoS One. 2024 Aug 29;19(8):e0309192. doi: 10.1371/journal.pone.0309192 (PMC11361592; doi:10.1371/journal.pone.0309192)
Supplement: S1 Fig — A = Wild type, B = Tom-Bd. Scale bars = 200 μm. (DOCX) [file pone.0309192.s001.docx]

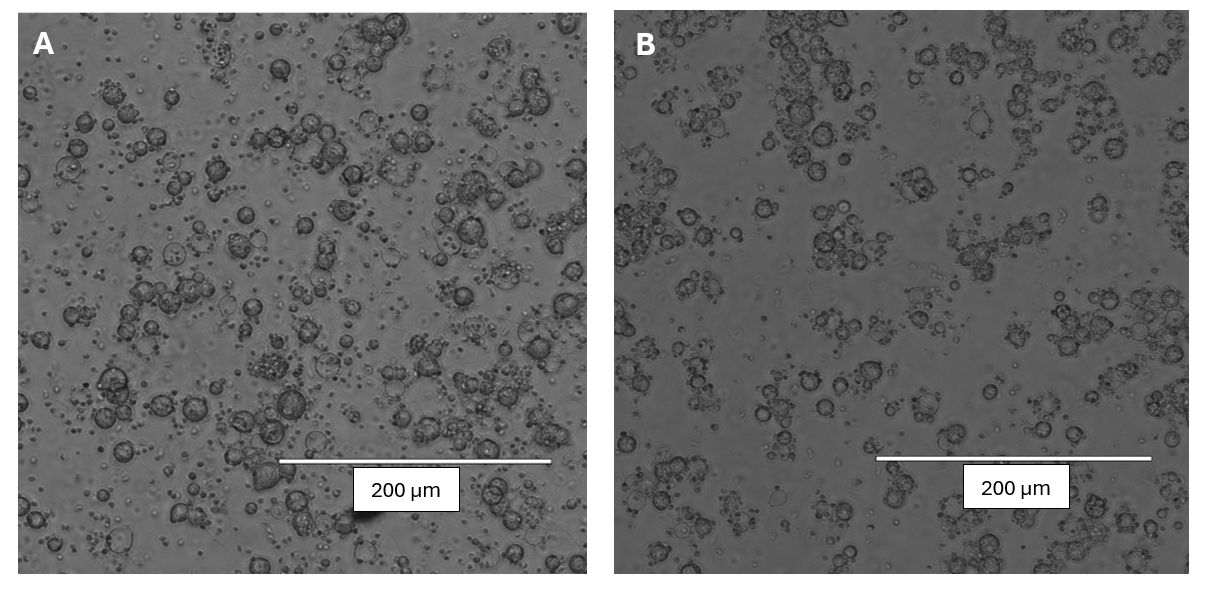


**Figure S1**-Growth of wildtype and transformed (Tom-Bd) Bd after 72 h in TGhL. A= Wild type, B= Tom-Bd. Scale bars = 200 µm
