# Supplementary material for: Stable in vitro fluorescence for enhanced live imaging of infection models for Batrachochytrium dendrobatidis
Source: PLoS One. 2024 Aug 29;19(8):e0309192. doi: 10.1371/journal.pone.0309192 (PMC11361592; doi:10.1371/journal.pone.0309192)
Supplement: S2 Fig — Both wildtype and Tom-Bd produced similar effects on host cells at 96 h post infection. A = Uninfected A6 cells, B = WT infected A6 cells, C = Tom-Bd infected A6 cells. D = Uninfected DWJ cells, E = WT infected DWJ cells, F = Tom-Bd infected DWJ cells. Scale bars = 200 μm. (DOCX) [file pone.0309192.s002.docx]

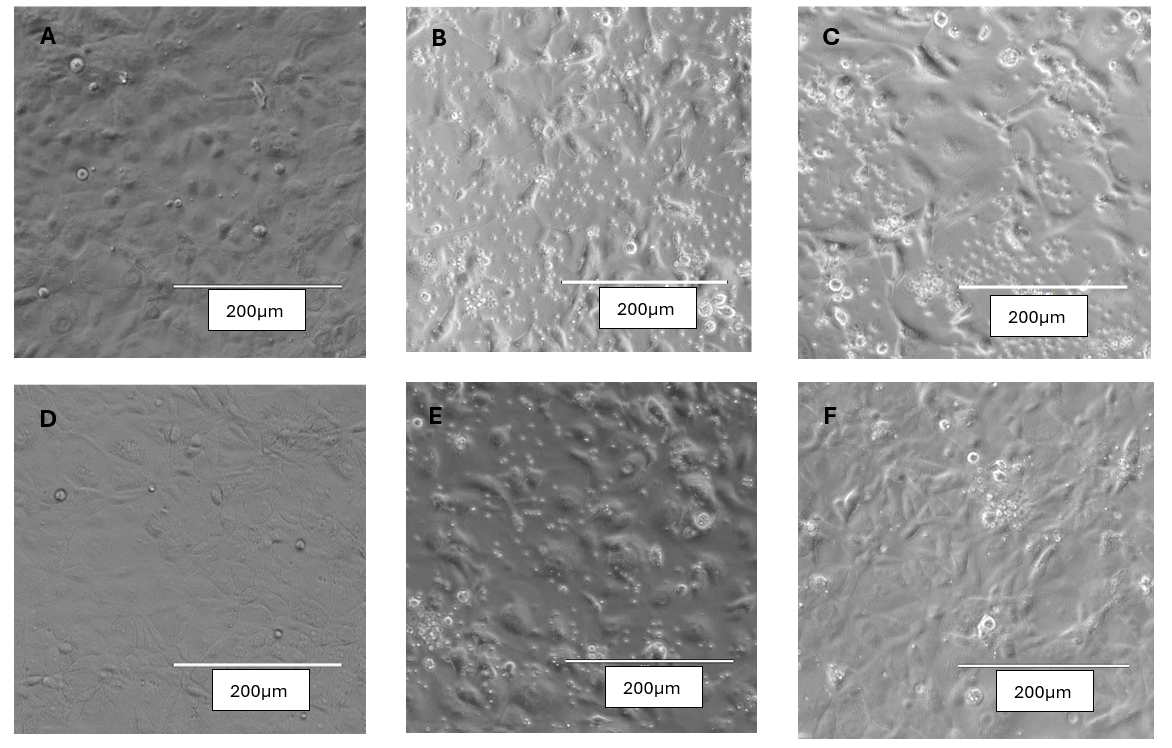


**Figure S2** Growth of wildtype or transformed Bd in host cells. Both wildtype and Tom-Bd produced similar effects on host cells at 96 h post infection. A= uninfected A6 cells, B= WT infected A6 cells, C= Tom-Bd infected A6 cells. D= uninfected DWJ cells, E= WT infected DWJ cells, F= Tom-Bd infected DWJ cells. Scale bars = 200 µm
