# Supplementary material for: Stable in vitro fluorescence for enhanced live imaging of infection models for Batrachochytrium dendrobatidis
Source: PLoS One. 2024 Aug 29;19(8):e0309192. doi: 10.1371/journal.pone.0309192 (PMC11361592; doi:10.1371/journal.pone.0309192)
Supplement: S3 Fig — A = 20°C, B = 24°C, C = 27°C. D = Number of mature zoosporangia in DWJ cells after 120 h incubation at different temperatures. Mature zoosporangia were counted per field of view (n = 15) at 20x. (DOCX) [file pone.0309192.s003.docx]

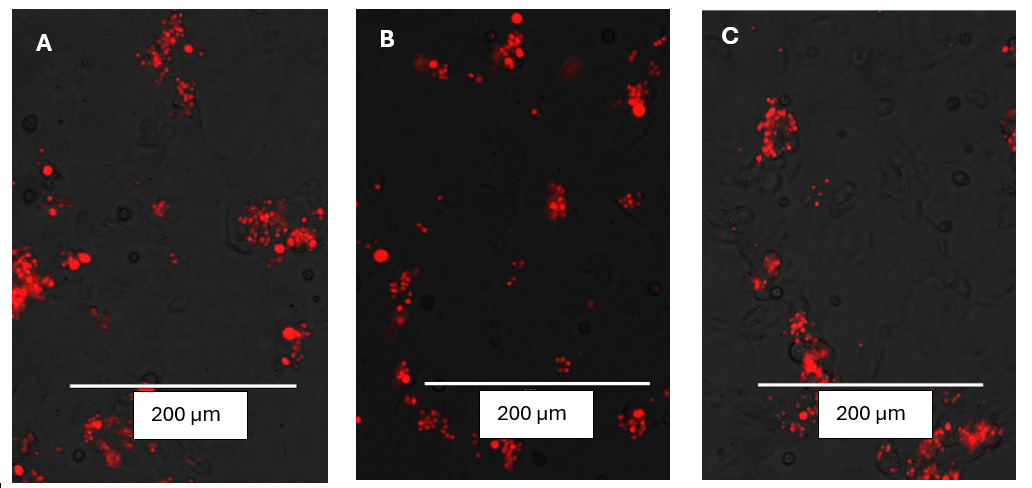


D

**Figure S3**-Growth of Tom-Bd in DWJ cells at different temperatures. A=20°c, B= 24°c, C= 27°c. D= Number of mature zoosporangia in DWJ cells after 120 h incubation at different temperatures. Mature zoosporangia were counted per field of view (n=15) at 20x.
